# Ultrasonography for Eating and Swallowing Assessment: A Narrative Review of Integrated Insights for Noninvasive Clinical Practice

**DOI:** 10.3390/nu15163560

**Published:** 2023-08-12

**Authors:** Keisuke Maeda, Motoomi Nagasaka, Ayano Nagano, Shinsuke Nagami, Kakeru Hashimoto, Masaki Kamiya, Yuto Masuda, Kenichi Ozaki, Koki Kawamura

**Affiliations:** 1 Nutrition Therapy Support Center, Aichi Medical University Hospital, Nagakute 480-1195, Japan; 2 Department of Geriatric Medicine, Hospital, National Center for Geriatrics and Gerontology, Obu 474-8511, Japan; 3 Department of Rehabilitation Medicine, Hospital, National Center for Geriatrics and Gerontology, Obu 474-8511, Japan; nagasaka-m@ncgg.go.jp (M.N.); khashi@ncgg.go.jp (K.H.); masasasasaki0404@yahoo.co.jp (M.K.); yuuto-m@ncgg.go.jp (Y.M.); ozk-kety@kyf.biglobe.ne.jp (K.O.); kawamura@ncgg.go.jp (K.K.); 4 Department of Nursing, Nishinomiya Kyoritsu Neurosurgical Hospital, Nishinomiya 663-8211, Japan; aya.k.nagano@gmail.com; 5 Department of Speech Language Pathology and Audiology, Faculty of Rehabilitation, Kawasaki University of Medical Welfare, Okayama 701-0193, Japan; nagami@mw.kawasaki-m.ac.jp

**Keywords:** aspiration, dysphagia, swallowing muscle, swallowing function, ultrasound

## Abstract

Dysphagia is a syndrome of abnormal eating function resulting from a variety of causative diseases, and is associated with malnutrition. To date, the swallowing function has been difficult to examine without the use of invasive and expensive methods, such as the videofluorographic swallowing study or fiberoptic endoscopic evaluation of swallowing. In recent years, progress has been made in the clinical application of ultrasound equipment for the evaluation of body compositions near the body surface, including the assessment of nutritional status. Ultrasound examination is a noninvasive procedure and relatively inexpensive, and the equipment required is highly portable thanks to innovations such as wireless probes and tablet monitoring devices. The process of using ultrasound to visualize the geniohyoid muscle, digastric muscle, mylohyoid muscle, hyoid bone, tongue, masseter muscle, genioglossus muscle, orbicularis oris muscle, temporalis muscle, pharynx, esophagus, and larynx, and the methods used for evaluating these structures, are provided in this study in detail. This study also aims to propose a protocol for the assessment of swallowing-related muscles that can be applied in real-world clinical practice for the diagnosis of sarcopenic dysphagia, which can occur in elderly patients with sarcopenia, and has received much attention in recent years.

## 1. Introduction

Dysphagia increases the risk of malnutrition, dehydration, aspiration pneumonia, and even death [1,2]. These complications delay functional recovery and reduce the quality of life if the patient is unable to eat or drink [1,2,3]. Previous reviews have highlighted that the videofluorographic swallowing study and fiberoptic endoscopic evaluation of swallowing can accurately identify dysphagia compared with clinical evaluation [1]. The aspiration of oropharyngeal secretions, subclinical aspiration (the absence of coughing response when food or liquid enters the airway), etc., can be observed during these procedures [4]. However, the videofluorographic swallowing study involves exposure to radiation and travelling to the examination room; meanwhile, fiberoptic endoscopic evaluation of swallowing causes pain due to the insertion of a probe into the pharynx through the nasal cavity. The available professional resources that can be used as a guide for performing these examinations are limited. As a result, nonhospital facilities have poor access to the resources required to perform the videofluorographic swallowing study and fiberoptic endoscopic evaluation of swallowing.

Speech–language–hearing therapists (SLHTs) and nurses, etc., who specialize in evaluating the swallowing function perform dysphagia screening tests. The different methods that can be performed at the bedside or in a typical eating scene include tests to identify patients at high risk for developing aspiration and other problems [5]. However, only a limited number of screening methods have been examined as the gold standard tests for assessing the risk of aspiration [6,7]. For example, the water swallowing test [8,9], Toronto bedside swallowing screening test [10], Gugging swallowing screen [11], and the volume–viscosity swallow test [12] are frequently used at the bedside to assess the swallowing function. Although a variety of tests and methods have been developed to assess the swallowing function, no highly accurate, noninvasive method has been established to assess swallowing function that SLHTs and nurses can easily use at the bedside.

In recent years, echography of the swallowing-related muscles has attracted research attention. It is one of the methods used for visualizing the swallowing movement. In general, echography is a widely used examination method in daily practice. It can be utilized to assess the skeletal muscles [13]. Recently, sarcopenia has been associated with dysphagia [14,15,16,17], and measurement of the swallowing-related muscle is clinically important in assessing the swallowing function. Despite its widespread use, promising potential, and over two decades of application in swallowing research, echography has not yet achieved broad recognition as a representative advancement in SLHT literature. This might be attributed to the ongoing debates concerning its efficacy and usage [18,19]. Previous systematic reviews and meta-analyses have evaluated the risk of aspiration and presence of pharyngeal residue associated with echography of the swallowing-related muscles [20]; the majority of these studies targeted specific subsets of swallowing-related muscles. The aim of this research is to extract and synthesize the insights from these specialized studies, providing an overview of how various swallowing-related muscles are visualized and evaluated using echography, and to shed light on the current status, challenges, and future prospects of echography in the assessment of swallowing function. In this regard, we reviewed the current use of echography for measuring muscle mass and assessing swallowing function, after determining the evaluation method suited for each observation site across all vocal and speech organs. This study does not apply specific statistical methods; instead, it is based on an exhaustive review of existing literature. Article searching was conducted in MEDLINE via the PubMed interfaces. Hand searching was also performed. The keywords used during the literature search were “(Ultrasonography OR Ultrasound) AND (swallowing OR dysphagia).” Journal articles written in English published from database inception until October 2021 were searched. 

Although the use of ultrasound technology is not universally authorized for all healthcare professionals, in certain regions, a diverse group of healthcare practitioners, including SLHTs and registered dietitians, are permitted to use ultrasound devices in clinical settings. Consequently, this study provides pertinent insights not only for practicing healthcare professionals, but also for students aspiring to become SLHTs and physicians who specialize in evaluating and managing swallowing disorders and using imaging techniques. Therefore, the potential reach of this study extends beyond geographic and professional boundaries. It fosters anticipation for the broader acceptance of these noninvasive assessment techniques in the global healthcare sector. This research serves to collate and enhance the collective knowledge derived from these specialized studies, thereby fortifying the potential for the application and widespread use of echography in routine clinical practice.

## 2. Geniohyoid Muscle (Figure 1)

The geniohyoid muscle is a short, slender, ribbon-shaped muscle originating from the inferior genial tubercle and inserted into the body of the hyoid bone. The two geniohyoid muscles on either side of the midline are parallel and close to each other [21]. It belongs to the suprahyoid muscle group, elevating the hyoid bone and lifting the oral floor during swallowing; the geniohyoid muscle moves the hyoid bone forward [21]. Decreased cross-sectional area, muscle thickness, and the brightness of the geniohyoid muscle during an ultrasound have been associated with swallowing function [22], sarcopenic dysphagia [23], and jaw opening strength [24]. Ultrasound is also used to determine the effects of swallowing exercises [25,26]. In addition, it is employed to evaluate the geniohyoid muscle movement during swallowing [27,28,29]. Ultrasound measurements of the morphology and movement of the geniohyoid muscle may be useful in assessing the swallowing function and determining the effectiveness of therapeutic interventions.

**Figure 1:**
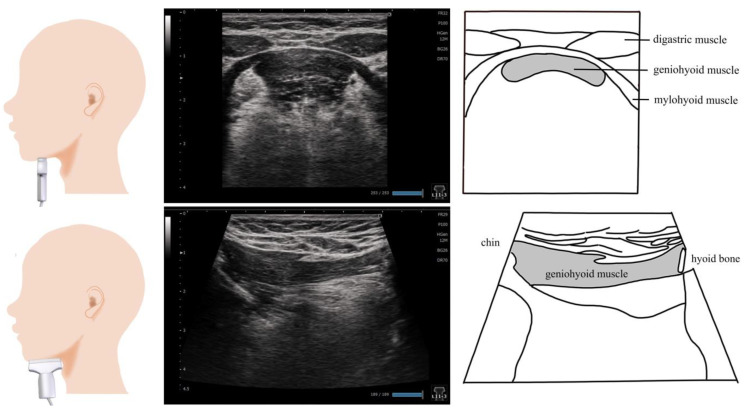
**Imaging of the geniohyoid muscle**. On the coronal section, the morphology of the geniohyoid muscle changes depending on the position of the probe. The digastric muscle and surficial mylohyoid muscle are appropriate locations to insert the probe. On the sagittal section, the probe should ideally be placed on the midline to visualize the hyoid bone and chin bone in one screen.

The method of applying the probe varies depending on the purpose of the measurement (Figure 1). The cross-sectional area of the geniohyoid muscle is measured in the coronal section using a linear probe [24,30]. The probe is placed vertical to the muscle fiber between the chin and hyoid bone [22,23,24,25,27,28,31,32,33]. The probe should be placed vertical to the chin plane. For the cross-sectional area, the geniohyoid muscle is extracted from the area surrounded by the fascia, indicated by the hyperechoic line, and the area excluding the fascia is calculated as the cross-sectional area [23,24,25]. The position of the probe varies from the middle [25] to the posterior one-third [22,28] of the line connecting the mandible and the hyoid bone, and to the posterior one-third [23,24] of the line connecting the parotid gland and hyoid bone. The muscle length changes and the contraction velocity during swallowing are measured on a sagittal section using a convex probe. The probe is placed horizontal to the muscle fibers [29,30,34]. The measurement is usually performed in a sitting position with the head and neck supported [34]; it can also be performed in a natural position [25,27,31] or a 30° reclining position [23,30].

## 3. Digastric Muscle (Figure 2)

The digastric muscle is a spindle-shaped muscle that consists of the anterior and posterior bellies joined by an intermediate tendon. Although it is regarded as one muscle, the anterior and posterior bellies have different embryological origins and roles [21]. The anterior belly arises from the mandible (the digastric fossa near the junction of the mandible), while the posterior belly arises from the temporal bone (the papillary notch); both of these structures terminate at the hyoid bone via the intermediate tendon [21]. During eating, the anterior belly of the digastric muscle opens the lower jaw and brings the hyoid bone forward and upward during swallowing. The posterior digastric muscle assists in the lateral and posterior movement of the lower jaw. It belongs to the suprahyoid muscle group. It depresses the mandible when the hyoid bone is in a fixed position, and elevates the hyoid bone when the mandible is in a fixed position [21,35].

**Figure 2:**
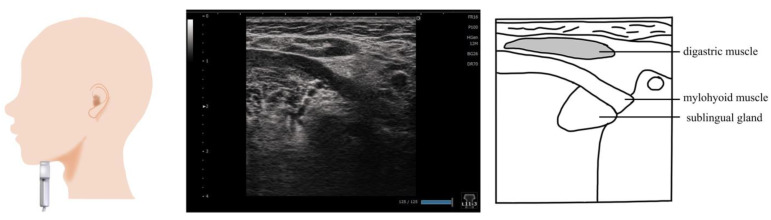
**Imaging of the digastric muscle**. The muscle belly is visualized by applying the probe vertical to the muscle fibers of the digastric muscle. Furthermore, the entire image is observed by sliding the probe from the chin to the top of the hyoid bone.

The probe is placed vertical to the fibers of the anterior belly of the digastric muscle, and the thickness is measured on the short axis (Figure 2). A linear probe is used for measurements [26,33,36]. The thickness of the digastric muscle is often measured as the distance from the top to the bottom border of the fascia at the thickest point vertical to the mylohyoid muscle [26,31,32]. The measurement is usually performed in a sitting position [31,32,37].

The thickness of the anterior belly of the digastric muscle is measured by ultrasound, and is used to determine the effects of muscle training, such as head lift exercises and expiratory muscle training, and kinesiology taping [25,31]. Although the ultrasound measurement of the digastric muscle is useful in determining the effects of therapeutic interventions, no study has reported its association with swallowing function; therefore, its relationship with swallowing function should be examined.

## 4. Mylohyoid Muscle (Figure 3)

The mylohyoid muscle is part of the suprahyoid muscle group that shapes the floor of the oral cavity. It works along with the geniohyoid muscle and the anterior belly of the digastric muscle, and aids in swallowing. It is also referred to as the accessory muscle of mastication as it is involved in mouth opening movements [38]. Previous studies have evaluated the therapeutic effects of maximal jaw opening exercises and head lift exercises, as well as tongue strengthening training, as part of dysphagia rehabilitation [26,31,32].

**Figure 3:**
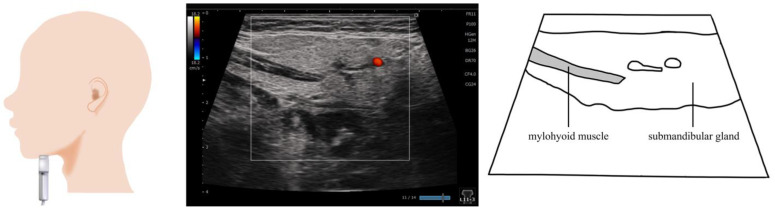
**Imaging of the mylohyoid muscle**. The probe is placed at the submental region and is visualized on a coronal section. When measuring muscle thickness, the probe should be placed in a position that allows the measurement of the thickness of the area where the submandibular gland wraps around the mylohyoid muscle.

The mylohyoid muscle is measured on the coronal section by placing a probe at the submental region (Figure 3). As the mylohyoid muscles run radially, the probe should be moved from front to back to visualize the largest and most distinct muscle [32,37]. Some previous studies reported that the mylohyoid muscle measurement area is the area directly below the measurement point of the digastric muscle, from the top to the bottom of the fascia [26,31,32]. However, in these studies, the point for visualizing and evaluating the mylohyoid muscle was ambiguous, and the submandibular gland was used as the orientation point when visualizing the mylohyoid muscle. Considering that the submandibular gland is a relatively homogeneous organ, the mylohyoid muscle imaged under the submandibular gland should be measured. The measurements are frequently performed in a sitting position [31,32,36].

## 5. Hyoid Bone

The hyoid bone is derived from the branchial arch, with no articulation to other bones. The suprahyoid muscle group (mylohyoid muscle, digastric muscle, stylohyoid muscle, and geniohyoid muscle) and the sublingual muscle group (sternohyoid muscle, omohyoid muscle, sternum thyroid muscle, and thyrohyoid muscle) terminate at the hyoid bone. When the jaw is opened, it is fixed downward by the sublingual muscle group; during swallowing, it is pulled toward the lower jaw by the contraction of the suprahyoid muscle group and moves upward and forward. The hyoid bone can be easily identified on an ultrasound image; ultrasound is useful for evaluating the laryngeal movement during swallowing by measuring the movement of the hyoid bone, travel distance, and duration of hyoid bone movement [37,39,40,41,42,43,44,45,46,47,48,49,50,51,52,53]. In comparison with healthy individuals, the laryngeal elevation distance is decreased in patients with neurologic disease who have dysphagia; moreover, the ratio of the hyoid [48] and laryngeal elevation distance is greater in stroke patients with dysphagia [39]. The ultrasonographic evaluation of hyoid–larynx approximation has good reliability [47]. Cergan et al. reviewed the measurement of the laryngeal structure via ultrasound, and proposed the utility of ultrasound as a safe alternative to endoscopy for evaluating the larynx in patients with coronavirus disease 2019 [54].

In most studies, the hyoid bone was measured by placing the probe vertical to the submental region on the midsagittal plane; in a previous study, the probe was placed on either the left or right side of the thyroid cartilage to avoid interfering with the swallowing movements [41]. The measurements are frequently conducted in a sitting position. When the measurements obtained with the manually operated (handheld) probe were compared with those obtained with the probe fixed in a stabilizer, a significant variability was observed in the measured values [43]. Good intra- and inter-rater reliabilities were also shown when the probe was covered with a self-designed water bag to provide good contact between the skin and probe [44]. During the visualization of the larynx and pharynx, including the hyoid bone, a condom filled with jelly was used for ultrasonography; then, the probe was covered to minimize the inhibition of swallowing and maximize its contact with the skin.

## 6. Tongue (Figure 4)

The tongue is the largest among the swallowing-related muscles, consisting of a base and body, and is easily visualized via ultrasound. As the muscle fibers run in various directions, are soft, and have well-developed musculature, many studies have measured the muscle mass and movements of the tongue. Some previous studies observed a reduction in tongue movement and tongue muscle mass in older adults and those with sarcopenic dysphagia [23,55], and reported an association between tongue thickness and diadochokinesis [56]. In addition, tongue movement can be captured during swallowing [57]; the assessment of tongue movement is useful for the diagnosis of tongue protrusion [58] and the evaluation of oral function, especially in children [59,60]. By contrast, some studies reported difficulties in determining dysphagia during tongue movement [61].

**Figure 4:**
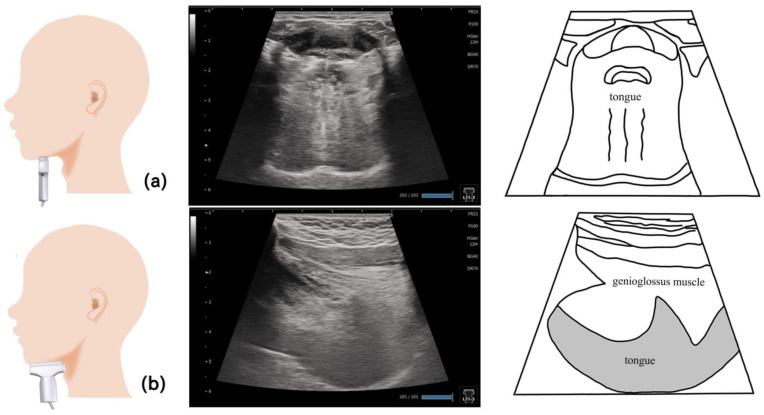
**Imaging of the tongue**. On the coronal section (**a**), the application of the probe to the submandibular surface vertical to the Frankfurt plane allows the visualization of the central part of the tongue. Ideally, the dorsum of the tongue should be visualized on a single screen by adjusting the depth. On the sagittal section (**b**), the entire tongue is visualized in the deep layer by applying the probe to the midline and visualizing the geniohyoid muscle and genioglossus muscle in one screen.

**Figure 5:**
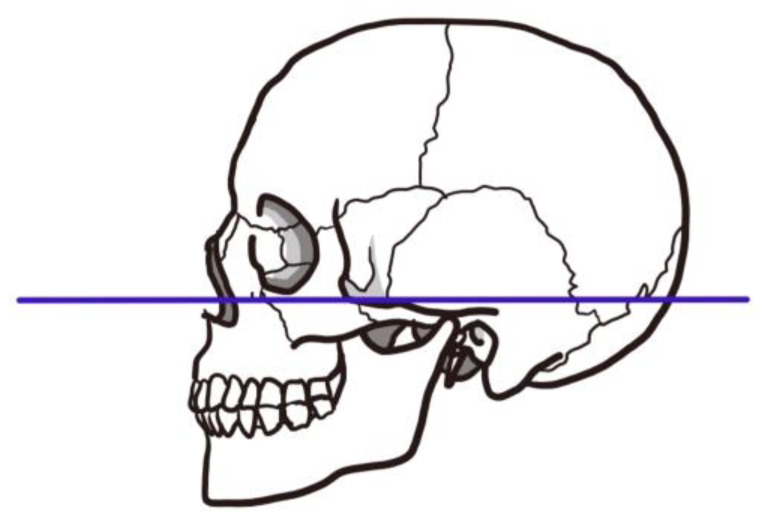
**Frankfurt horizontal plane**. The Frankfurt horizontal plane is a straight line connecting the superior margin of the ear canal and the inferior margin of the orbit. As it appears almost horizontal when viewed on a natural position, the plane determined by this line on the left and right portion is called the Frankfurt plane or ear–eye plane, and is used as the standard plane to determine the position and direction of the skull.

The postures and methods of positioning the tongue during ultrasonography differ (Figure 4). With regard to the posture, most of the previous studies used the sitting position [37,50,56,57,59,60,61,62,63], while some used the standing position [55] and supine position [23]. For the evaluation of the central part of the tongue, the probe is often placed vertical to the Frankfurt plane (Figure 5) on the submandibular surface [23,58,59,61]. On the base of the tongue, another method is used that involves applying the probe at a 45° angle to the Frankfurt horizontal plane in line with the second premolars [56]. Convex (3.5 MHz) [50,63] or sector (3.5–5 MHz) [57,58] probes are used when measuring the tongue. By contrast, some studies used the linear type (7.5 MHz) [23,33,37]. However, as the tongue is located in the deep layer, the linear type requires more time for gain and in-depth adjustment, and its imaging accuracy may not be high. Tongue thickness is assessed based on the distance from the mylohyoid muscle to the dorsum of the tongue on the coronal section [33,37,50,63]. During examination, the head and neck are positioned on the midline [60]; however, some studies used a holder [57,61] or headset [59] to secure the neck and jaw to accurately capture the tongue movement.

## 7. Masseter Muscle (Figure 6)

The masseter muscle is a relatively large swallowing-related muscle; it is located in an area that is not easily affected by the bone, making it suitable for ultrasonography. The association between decreased masseter muscle thickness and decreased masticatory function [64] and the risk of developing dysphagia [65] have been reported. However, the masseter muscle thickness can vary greatly depending on the measurement site [66]. Therefore, appropriate testing protocols should be established to ensure inter- and intra-rater reliability [67].

**Figure 6:**
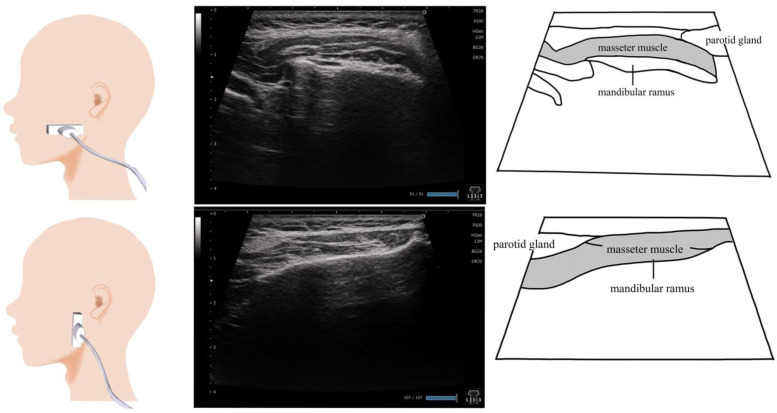
**Imaging of the masseter muscle.** To visualize the masseter muscle, the probe can be placed vertical to the muscle fibers or horizontal to the muscle fibers. The entire area, including the bone attachment site, is visualized, and the thickest site is measured. As the purpose of the examination is to evaluate the swallowing function, the examined patient should be placed in a sitting position and looking forward or slightly downward.

A linear probe is placed vertical to the muscle fibers of the masseter muscle to measure the thickness of this muscle (Figure 6). The thickest site of the visualized masseter muscle and its thickness are determined [64,66,68,69]. The thickness of the masseter muscle, visualized with a probe placed parallel to the muscle fibers, is easy to measure in the clinical setting [66]. To identify the site of maximal thickness, the entire masseter muscle should be uniformly observed down to the zygomatic bone and lower jaw attachment before determining the measurement site. The changes in the masseter muscle thickness and contractility should be assessed during a relaxed state with the mouth closed and teeth clenched [64,67]. Meanwhile, the trunk, head, and neck can be placed in an upright or natural position during examination [64,67,68]. 

## 8. Genioglossus Muscle (Figure 7)

The genioglossus muscle is the major extrinsic tongue muscle that makes up the body of the tongue. It is located at the lingual side of the geniohyoid muscle; it originates from the chin bone and terminates at the dorsum of the tongue and hyoid bone. The genioglossus muscle moves the tongue forward and downward. The genioglossus muscle is a respiratory-related muscle. During inspiration, the genioglossus muscle is displaced downward [70,71]. The genioglossus muscle is reportedly involved in airway obstruction during sleep apnea [72]. Kakudo et al. examined the inter-rater reliability of ultrasound assessment on the genioglossus muscle [73]. They reported that the thickness can be measured from either the upper or lower side of the fascia, depending on the examiner’s judgment, and is less reliable compared with the method used for measuring the geniohyoid muscle.

**Figure 7:**
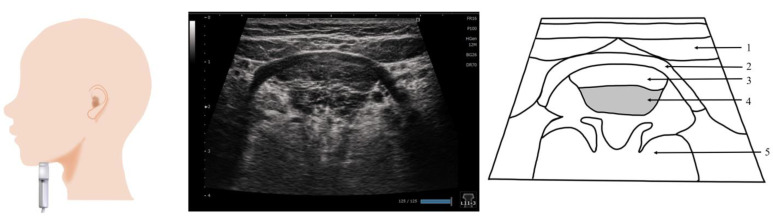
**Imaging of the genioglossus muscle**. The probe is placed vertical to the Frankfurt plane. As the genioglossus muscle partially reaches the dorsum of the tongue, the coronal section, with other muscles as the orientation point, may be a suitable standard evaluation image. 1, Digastric; 2, mylohyoid; 3, geniohyoid; 4, genioglossus; 5, the body of the tongue.

The sagittal and coronal sections are suitable for ultrasound imaging of the genioglossus muscles (Figure 7). Many studies that examined ultrasound images obtained from the sagittal sections have reported that the evaluations were performed using sector-type or convex-type probes [60,61,70,71,74]. Because the end of the genioglossus muscle reaches the dorsum of the tongue, a probe that allows deep observation may be preferred. On the sagittal section, the probe is placed on the midline of the upper hyoid bone. A linear probe was used in the imaging examination of the coronal section [60]. Therefore, the genioglossus muscle in the shallower layer can be observed. However, if it is viewed in a coronal section, the thickness of the genioglossus muscle and the area visualized vary depending on the position of the probe. A different position was proposed to visualize the global coronal section, with other muscles simultaneously visualized as orientation points. The preferred posture may be similar to that during eating, that is, a sitting position with the head facing forward [60,61,73,74]. 

## 9. Orbicularis Oris Muscle (Figure 8)

Because the orbicularis oris muscle is involved in articulation and swallowing, adequate muscle strength and endurance should be maintained. Reduction in the muscle mass of the orbicularis oris muscle causes leakage of food into the mouth during swallowing, and decreases the intraoral pressure during swallowing [75,76]. When the maximal strength and endurance of the orbicularis oris muscle in younger adults were compared with those in older adults, both maximal muscle strength and endurance of the orbicularis oris muscle were significantly lower in older adults [75]. The orbicularis oris muscle has a hierarchical structure with superficial muscles involved in articulation and deep muscles involved in swallowing. Zhang et al. examined the organization of the orbicularis oris muscle in healthy individuals and patients who underwent surgery for the presence of a cleft lip; the orbicularis oris muscle had a hierarchical structure with different superficial and deep muscle fibers [77].

**Figure 8:**
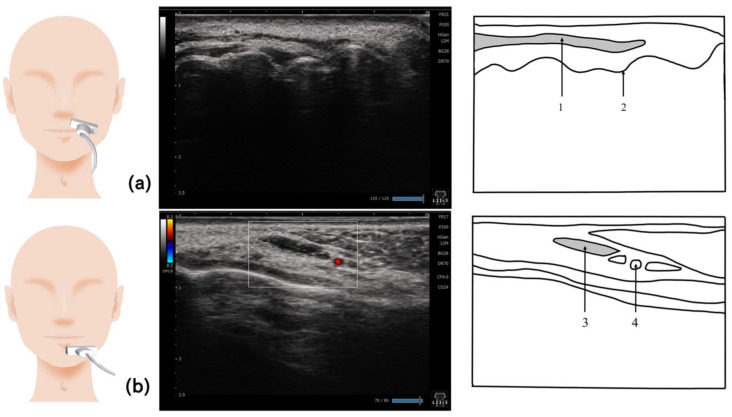
**Imaging of the orbicularis oris muscle.** The orbicularis oris muscle can be visualized by placing the probe along the margin of the upper lip (**a**). For the depressor anguli oris muscle, the probe should be placed vertical to the muscle fibers. Since this muscle is located just underneath the surface of the skin, a linear probe should be used (**b**). 1, Orbicularis oris muscle; 2, alveolar ridge; 3, depressor anguli oris muscle; 4, inferior labial artery.

The orbicularis oris muscle is measured by placing the probe on the short axis vertical to the nasal septum, following the curve of the upper lip along the margin, and moving the probe left and right from the midline, or by positioning the probe vertical to the philtrum using the short axis [77,78]. Ultrasonography of the orbicularis oris muscle measures the thickness of the superficial and deep layers after a cleft lip repair, clearly demonstrating the hierarchical structure of the orbicularis oris muscle [77]. Ultrasonography is useful for visualizing the target areas during surgical treatments involving the lips, such as cleft lip and facial paralysis, which are difficult to confirm by inspection alone [78,79]. Facial muscle is a generic term used to describe the cutaneous muscles that are located in the superficial layer of the head and terminate at the skin on one side. Ultrasonography has been used to show the reference values of six facial muscles: frontalis, orbicularis oculi, orbicularis oris, depressor anguli oris, depressor labii inferioris, and mentalis muscles [80]. This study focused on examining the depressor anguli oris, one of the facial muscles surrounding the orbicularis oris muscle. Although there is no clearly defined method for visualizing the depressor anguli oris, visualization can be performed by applying the probe to the muscle fibers on the short axis (Figure 8). 

## 10. Temporalis Muscle (Figure 9)

The temporalis muscle is extensively located in the superficial layer among the swallowing-related muscles, and runs from the left and right temporal bones to the muscular processes of the mandible. The temporalis muscle is among the primary muscles of mastication, while the masseter muscle elevates the mandible and pulls it backward. The masseter muscle thickness and temporalis muscle thickness correlate with age, sex, and body mass index [67]. As the muscle activity of the temporalis muscle decreases to the extent that the dentition can collapse, the muscle activity of people wearing complete dentures is significantly lower than that of people with natural dentition [81]. Following the standardized protocols, ultrasound can be used to assess the superficial and deep masticatory muscle thickness. However, as the target population is limited, patients with clinical conditions such as temporomandibular disorders, malalignment, and dysphagia should be examined.

**Figure 9:**
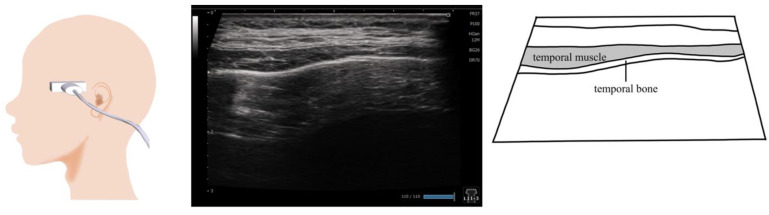
**Imaging of the temporalis muscle**. The probe is placed horizontally against the superior margin of the zygomatic arch and is moved gradually in a cephalic direction toward the temporal fossa. When evaluating muscle mass, the thickest site should be measured.

Temporalis muscle thickness refers to the maximum distance between the lateral and medial fascia. The probe is placed at the superior margin of the zygomatic arch, and the depth is set at 40 mm. Then, the probe is moved gradually toward the head parallel to the short axis of the zygomatic arch and toward the temporal fossa until the temporalis muscle appears on the screen (Figure 9). During measurement, the patient should sit comfortably on a chair with a backrest; the measurement is conducted at the left and right sides when the muscles are relaxed, the teeth are clenched, and the mouth is opened wide. The patient is instructed to clench the teeth as forcefully as possible without inducing pain in the teeth. The patient is then instructed to open the mouth as widely as possible without causing discomfort to the temporomandibular joint [67].

## 11. Pharynx (Figure 10 and Figure 11)

**Figure 10:**
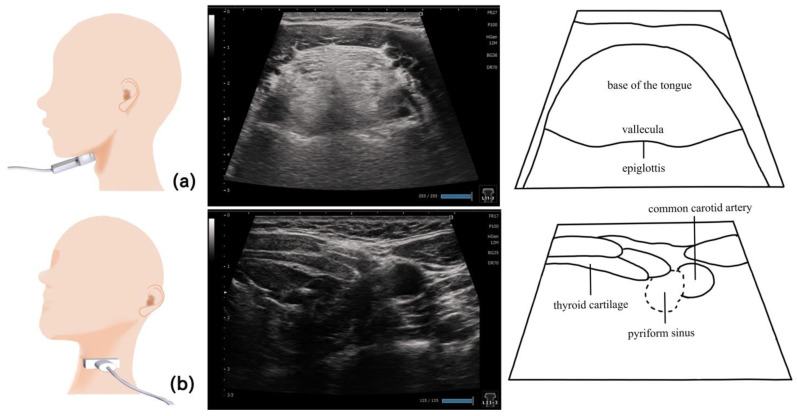
**Imaging of the pharynx**. To visualize the epiglottic vallecula, the probe should be placed horizontally at the level of the hyoid bone, so that the base of the tongue and the epiglottis can serve as the orientation points and the epiglottic vallecula can be identified (**a**). To visualize the left pyriform sinus, the probe should be placed horizontally from the left side at the level of laryngeal prominence (**b**). The thyroid cartilage and common carotid artery serve as the orientation points.

**Figure 11:**
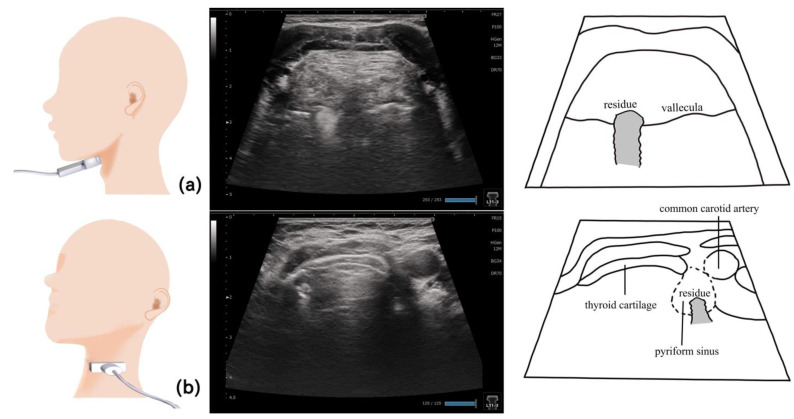
**Pharynx in a dysphagic patient**. A dysphagic patient showing residues in the vallecula (**a**) and pyriform sinus (**b**) on the ultrasound image.

The pharynx is a tubular space extending from the base of the skull behind the nasal cavity to the superior esophageal sphincter. Patients with pharyngeal residue have a 2.8-fold risk of aspiration compared with those without pharyngeal residue [82]. Ultrasonography allows the detection of the residues in the pyriform sinus and epiglottic vallecula (Figure 10 and Figure 11) [82]. Echography was used to evaluate the lateral pharyngeal wall motion during various swallowing maneuvers (supraglottic, super-supraglottic, and Mendelsohn maneuvers) [83].

Visualizing the pharynx with echography allows the detection of the residues in the pyriform sinus and epiglottic vallecula [82]. To visualize the left pyriform sinus, the probe is placed horizontally on the left side at the level of the laryngeal prominence, avoiding the midline. The common carotid artery and thyroid cartilage become the orientation points, and the pyriform sinus can be visualized by swinging the probe as necessary. The epiglottic vallecula can be visualized by placing the probe horizontally at the midline at the level of the hyoid bone, establishing the tongue root and epiglottis the orientation points and allowing the epiglottic vallecula to be visualized. Residue can be observed as a hyperechoic area or point (Figure 11). The ideal positioning during examination is the same as that during eating. The head and neck are not fixed to allow the patient to easily swallow at the most comfortable position [82]. Other methods include fixing the head and neck with a head stabilizer [84] and using two probes to evaluate the lateral pharyngeal wall motion [83].

## 12. Esophagus (Figure 12)

The esophagus is a tubular organ located at the foot of the pharynx, beginning at the level of the inferior margin of the cricoid cartilage; it moves the food mass from the pharynx to the stomach. At the upper end of the esophagus (the border between the pharynx and esophagus) is the superior esophageal sphincter; its incomplete closure causes pharyngeal reflux, aspiration, and pneumonia. The opening capacity of the esophagus increases as the food viscosity increases, suggesting that ultrasonography can be used to evaluate the opening of the esophagus and upper esophageal sphincter [85]. In terms of the water transit time, the front and rear edges of the image displayed during an ultrasound may lack sharpness depending on the food form and swallowing style. In a previous study, due to the slight differences in swallowing styles, the water transit times did not show similar trends among the examined patients [86].

**Figure 12:**
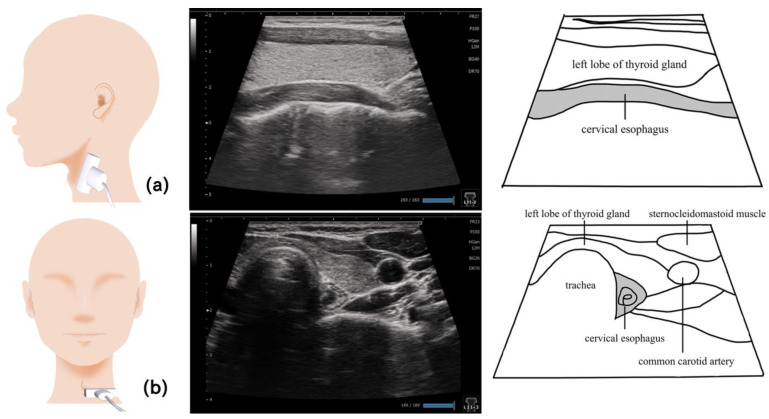
**Imaging of the cervical esophagus**. The cervical esophagus near the lower end of the cricoid cartilage is visualized by placing the probe longitudinal to the esophagus (**a**). Visualizing the thyroid gland on the surface of the esophagus provides clear images with less artifacts and noise. In the horizontal section, the esophagus can be visualized deeper than the thyroid gland, on the side of the trachea (**b**).

During examination, the patient should be seated vertically so that the Frankfurt horizontal plane is parallel to the floor. The inferior margin of the cricoid cartilage is used as the orientation point; the probe is directed longitudinally from the left toward the esophagus to obtain an image of the opening of the cervical esophagus located 1 cm closer to the foot from the orientation point [85,87]. Tilting the probe in the sagittal and forehead directions allows the visualization of the esophagus opening [84]. The function of the esophagus opening in patients with dysphagia can be evaluated by measuring the opening capacity and the duration of esophagus opening depending on the food consumed [85,86] (Figure 12). 

## 13. Larynx (Figure 13)

The larynx vibrates the vocal cords to produce speech and prevents foreign substances from entering the airways. Foreign body entry into the larynx or aspiration can be observed by performing a videofluorographic swallowing study or swallowing endoscopy. In recent years, foreign bodies were visualized using ultrasound [88,89,90]. By evaluating the risk of aspiration during meals using an echocardiogram, the effect of the compensatory swallowing method and food form adjustment were confirmed to reduce aspiration [88,89,90].

**Figure 13:**
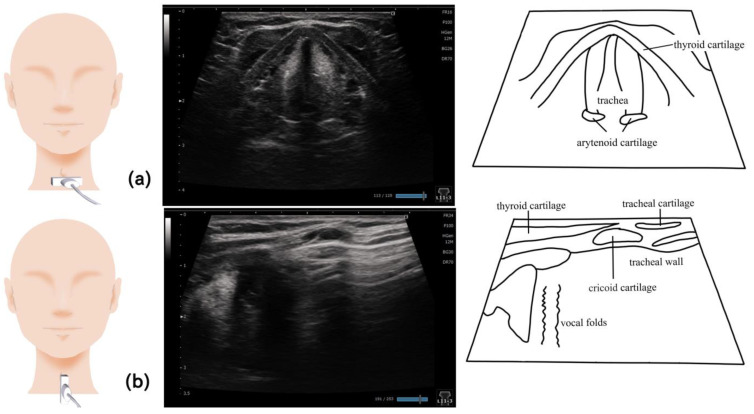
**Imaging of the larynx**. When imaging on a horizontal section, the probe is placed at the level of the thyroid cartilage; the vocal fold and arytenoid cartilage can be visualized when the patient speaks (**a**). On the sagittal section, the vocal cords, trachea, and trachea wall can be visualized by placing the probe on the midline of the thyroid cartilage (**b**).

The larynx is mainly visualized at the level of the thyroid cartilage, with the probe placed on a short axis (Figure 13). Many studies have identified the vocal cords by asking the examined patients to speak [89,91,92]. The probe was also placed on the sagittal section against the thyroid cartilage and moved to the back and then to the foot part to visualize the arytenoid cartilage [92]. The position required for ultrasonography varies. Some studies used a comfortable posture (similar to that during eating) without fixing the head and neck to avoid interfering the swallowing movements [88,89]; other studies used supine position with the head and neck extended [91,92,93]. The posture assumed during eating is recommended when undergoing laryngeal examination [89,90].

The aspirated material can be evaluated by placing the probe on the midline of the thyroid cartilage longitudinally to visualize the vocal cords and trachea wall. The structure of the trachea wall must be assessed prior to the examination to determine the presence of aspirated materials. The flow of aspirated material can be observed after swallowing as the movement that differs from the structures, a foggy hyperechoic area above the vocal fold, and a hyperechoic area on a line along the trachea wall in the direction of the trachea area [82,88,89].

## 14. Swallowing Function Evaluation

Observation of the tongue, pharynx, larynx, esophagus, and hyoid bone is useful when evaluating the swallowing function with an echographic device. By observing the tongue, its movement can be evaluated during swallowing. By observing the pharynx, the residues of the pyriform sinus and epiglottic vallecula can be evaluated. By observing the lateral pharyngeal wall motion, the supraglottic and Mendelsohn maneuvers can be evaluated. The capacity of the esophageal opening can be evaluated by measuring the opening capacity and the duration of opening. Aspiration can be visualized by assessing the larynx. Observing whether aspiration occurs during a meal allows the evaluation of the compensatory swallowing methods and food forms. Measurement of the hyoid bone movement, travel distance, and travel time is useful for evaluating the laryngeal movement during swallowing. They are usually evaluated during visualization procedures, but many studies examine these factors subjectively.

Evaluation of dysphagia associated with sarcopenia requires muscle mass assessment of the swallowing-related muscles [15]. Table 1 shows a practical echographic protocol for evaluating the swallowing-related muscles. During muscle mass evaluation, the static images can be used as objective measurements. The standard values are expected to be reported.

## 15. Summary

We clarified the latest findings on the method used for conducting an ultrasound assessment of the swallowing function. Several skeletal muscles and organs that are involved in swallowing can be evaluated by echography, both dynamically and statically. Visualization of the pharynx and larynx enables the detection of aspiration and residues. The swallowing function can be evaluated by measuring the travel distance and duration of hyoid bone movement and the opening capacity and the duration of esophagus opening. Many studies have shown the associations between swallowing-related skeletal muscle mass and swallowing function measured by echography; the results indicate that echography is the preferred method for evaluating the swallowing function.

Several issues need to be addressed in the use of echography for the evaluation of the swallowing function. First, standard guidelines on the position and orientation of the probe when measuring a certain site have not yet been established. Depending on the muscle or organ, orientation points should be set for proper visualization. Second, the posture during measurement is not consistent. In most cases, the measurements were obtained in a sitting position; the neck angles varied, from a neutral posture (assumed during eating) to an extended posture. This finding implies that evaluation should be carried out in a position that is the same as that assumed during eating. Another issue is that the probe is not stable due to the swallowing movements; hence, a holder or probe cover is sometimes used to hold the probe in place. Third, only a few studies that conducted food-based assessments were published. In order to effectively apply echography for the evaluation of swallowing function in clinical practice, the methods used for assessing the swallowing movement and function should be standardized. 

Echography is expected to be clinically applicable as a noninvasive method for assessing the swallowing function at a patient’s bedside. Muscle mass is an important factor in nutritional evaluation, and an echocardiogram is used as a nutritional evaluation device [94,95,96]. Echocardiography allows the evaluation of the swallowing-related skeletal muscles to identify any disorders, such as sarcopenia dysphagia, which are difficult to visualize using conventional methods. Technological advancements have been made, making the echocardiogram a portable and easy-to-use device. The use of artificial intelligence will enable the accurate identification of sites to be visualized and abnormal findings. 

## Figures and Tables

**Table 1:** Protocol for evaluating the swallowing muscle mass in clinical practice.

Muscle	Methods			
	Posture	Probe	Position and Orientation	Evaluation Method
Geniohyoid cross-sectional area/thickness	Sitting position	Linear	Sagittal section	Identify the area surrounded by fascia and calculate the cross-sectional area. Measure the muscle thickness.
Digastric cross-sectional area/thickness	Sitting position	Linear	Vertical to muscle fibers	Calculate the cross-sectional area. Measure the muscle thickness.
Mylohyoid thickness	Sitting position	Linear	Coronal plane	Measure the thickness of the mylohyoid muscle under the submandibular gland.
Tongue thickness	Sitting position	Convex	Coronal plane	Measure the distance from the mylohyoid muscle to the dorsum of the tongue.
Masseter thickness	Sitting position	Linear	Vertical to muscle fibers	Measure the thickest site.
Depressor anguli oris	Sitting position	Linear	Vertical to muscle fibers	Calculate the largest area.

## Data Availability

Not applicable.
